# Bamboo Plantations for Phytoremediation of Pig Slurry: Plant Response and Nutrient Uptake

**DOI:** 10.3390/plants9040522

**Published:** 2020-04-17

**Authors:** Julien Piouceau, Frédéric Panfili, Grégory Bois, Matthieu Anastase, Frédéric Feder, Julien Morel, Véronique Arfi, Laurent Dufossé

**Affiliations:** 1 PHYTOREM S.A., site d’Areva, chemin de l’autodrome, 13140 Miramas, France; j.piouceau@hotmail.fr (J.P.); fred_panfili@yahoo.fr (F.P.); boisgregory@gmail.com (G.B.); matthieu.anastase@gmail.com (M.A.); veroniquearfi@phytorem.com (V.A.); 2 CHEMBIOPRO Chimie et Biotechnologie des Produits Naturels, ESIROI agroalimentaire, Université de La Réunion, 15 avenue René Cassin, 97400 Saint-Denis, Réunion Island, France; 3 CIRAD, UPR Recyclage et Risque, F-34398 Montpellier, France; frederic.feder@cirad.fr (F.F.); julien.morel@cirad.fr (J.M.); 4 Recyclage et Risque, Université de Montpellier, CIRAD, F-34398 Montpellier, France

**Keywords:** bamboo species, pig slurry, biomass yield, wastewater treatment

## Abstract

On Réunion Island, a French overseas territory located in the western Indian Ocean, increasing pig livestock farming is generating large quantities of slurry. Most of it is spread on a little agricultural land due to the insular context. Considering the limitation of the quantities that can be spread on agricultural areas (European “Nitrate Directive” 91/676/EEC), the use of wastewater treatment systems using phytoremediation principles is an attractive option for the pig slurry treatment. A wastewater treatment system using bamboo groves was assessed for the pig slurry treatment. Three field plots were designed on an agricultural area and planted with 40 bamboo clumps on each plot. A total of 67 m^3^ of pig slurry was spread on two plots in two forms: raw slurry and centrifuged slurry. The latter plot was watered with tap water. The total amount of nitrogen, phosphorus and potassium was 5.3, 1.4 and 5.5 t·ha^−1^, respectively, for the raw slurry treatment and 4.2, 0.4 and 5.1 t·ha^−1^, respectively, for the centrifuged slurry treatment. The response of bamboo species to pig slurry application was determined using morphologic parameters, Chlorophyll *a* fluorescence measurements and biomass yield. Compared to the control, the biomass increased by 1.8 to 6 times, depending on the species and the form of slurry. Depending on the species, the average biomass ranged from 52 to 135 t.DM.ha^−1^ in two years of experiment.

## 1. Introduction

With the increase of intensive agriculture and livestock farming, large quantities of manure are generated [1]. Most of this manure is spread on agricultural land as a source of nutrient for crops [2]. However, such practice causes environmental problems due the over-application of animal manure to soil [3]. This over-application generates nitrate and phosphorus leaching [4] into groundwater that causes eutrophication, algae proliferation and acidification [5,6,7]. Moreover, dissolved organic matter and fecal bacteria [8] are increased in river and sea water bathing area [9,10]. For example, in French Brittany, high quantities of algae cover the sand and rot on the beach that causes nuisance smells detrimental for tourism.

On the Réunion island, a French and European overseas territory, livestock farming has been increasing since the 1980s. The livestock effluent production represents an amount of 636,000 tons per year, corresponding to 2325 tons of nitrogen [11,12]. The problem is worse on this island, due to the shortage of land suitable for spreading. Indeed, high quantities of pig manure are spread on agricultural land, up to 1.5 t·ha^−1^·yr^−1^ [13,14] or are spread illegally into gullies [11]. These practices in addition to the overflows of wastewater treatment plant and the anthropogenic input from septic tank lead to an enrichment of the nitrate and phosphorus content of the groundwater and sea-water’s coral reef causing the slow death of the coral and proliferation of algae [15,16,17].

Considering the limitation of the quantities that can be spread on agricultural area (European “Nitrate Directive” 91/676/EEC), the use of wastewater treatment systems using phytoremediation principles emerged as an attractive option for the treatment of manure [18]. Over the last decades, wastewater treatment systems using phytoremediation principles have been developed [19,20]. Most of these features are constructed wetland based on the use of aquatic plants [21]. Another type of system uses terrestrial plants like bamboo to treat wastewater [22,23,24,25]. This wastewater treatment technology can help to reduce the surface area for animal manure spreading and are able to treat the effluent directly on the field near the livestock farm, avoiding any transport. As a plant for treating wastewater, bamboo is interesting in many respects. The bamboo species’ dense root system favors the rhizodegradation [20] of organic compounds contained in wastewater. With regards to the biomass yield of aboveground parts, a bamboo plantation can be sized to effectively remove nutrients carried by wastewater. Moreover, mature bamboo plantations have high evapotranspiration rates [26,27] allowing high volumes of wastewater to be spread on plantations. By selecting the species that are most adapted in terms of wastewater nutrient removal, growth rate and biomass yield, it may be possible to reduce the surface area, thus optimizing the treatment system. Moreover, the medium and giant species of bamboo have a high growth rate and a high biomass yield [28,29,30] creating a high value-added for livestock farmer. Indeed, the bamboo biomass can be use as wood for bioenergy [28] and as mulching [31] for agricultural land and livestock farming.

Bamboo represents over 70 genera and 1200 species in the *Bambusoideae* subfamily and is present all over the world [32]. All of these species show different growth rates and biomass yields that are highly specific to each species. The growth of bamboo is defined by the number of new shoots or culms, produced during one full year. Each year, the culms increase in number and size (diameter and height). In our study, the response of bamboo species to pig slurry supply was determined using the number of shoots produced during the experiment and their diameter; the biomass yield was also quantified. Chlorophyll *a* fluorescence measurements were taken to determine the photosynthetic activity of the bamboo species. The aim of this study was to quantify the nitrogen fate into the soil and the part which is uptake by bamboo species and obtain biomass yields in order to select the most suitable bamboo species for wastewater treatment.

## 2. Results

### 2.1. Effect of Pig Slurry Application on Bamboo Growth

**Figure 1:**
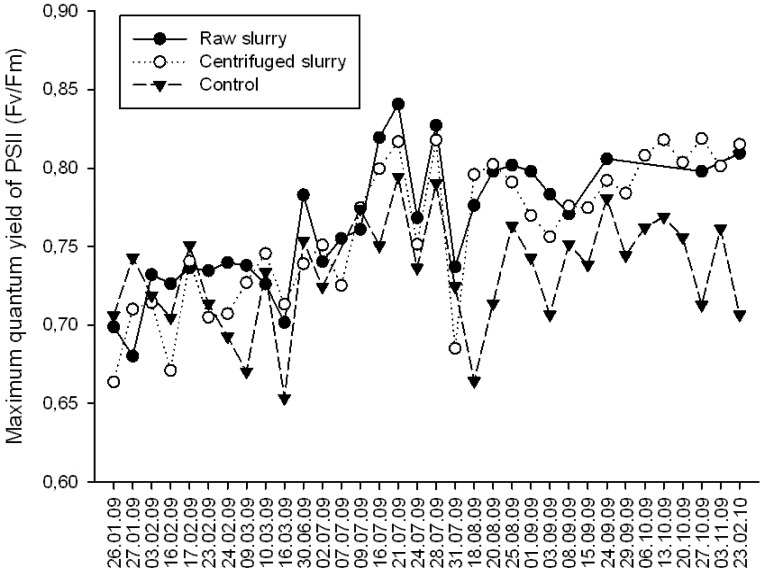
Average values of the maximum quantum yield of the Photosystem II (*Fv/Fm*) of the species *Gigantochloa wrayii* (GW), *Bambusa oldhamii* (BO), *Bambusa vulgaris* (BVV) by treatment plot (January 2009-February 2010 out of the total 2-year experiment: June 2008-June 2010). Chlorophyll fluorescence parameter *Fv/Fm*, which is the ratio of variable to maximum fluorescence after dark-adaptation, represents maximum quantum yield of PSII. The parameter has begun to be used for detecting stress in plants. In this study, *Fv/Fm* distribution pattern was analyzed in plants under various stress conditions in order to obtain basic knowledge for identifying the stress factor.

All bamboo species showed *Fv/Fm* ratios above 0.700 (Figure 1) for the two-pig slurry treatment plot. For the control plot, the *Fv/Fm* ratio showed value under 0.700, with an extreme low value of 0.653 in March 2009. The *Fv/Fm* ratios reach maximum value of 0.840 and 0.818 for the species planted on the raw slurry treatment plot and centrifuged slurry treatment plot, respectively. The *Fv/Fm* ratios were significantly higher for the bamboo species irrigated with pig slurry than those with tap water in the control plot (*p* < 0.001). Between the beginning and the end of the experiment the *Fv/Fm* ratio increased from 0.699 to 0.810 for the raw slurry treatment plot and from 0.664 to 0.815 for the centrifuged slurry treatment plot. The species from the control plot showed the same *Fv/Fm* ratios between the beginning and the end of the experiment.

**Figure 2:**
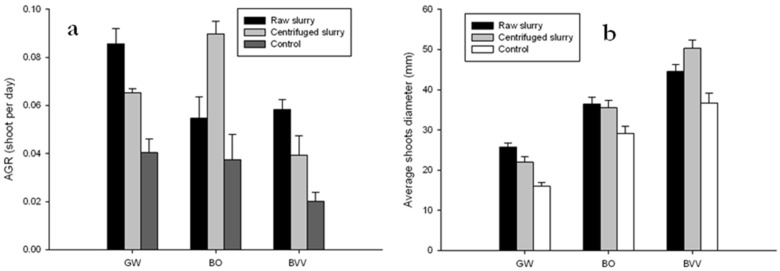
Average absolute growth rate (**a**) and average shoot diameter (**b**) by species and treatment plot; *Gigantochloa wrayii* (GW), *Bambusa oldhamii* (BO)*, Bambusa vulgaris* (BVV).

All bamboo species irrigated with pig slurry showed a significant increase of the shoots absolute growth rate (AGR) compared to the control plot (*p <* 0.001) (Figure 2a). For the species *Gigantochloa wrayii* (GW), the AGR was 0.04, 0.08 and 0.06 for the control, raw slurry and centrifuged slurry treatment, respectively. For the species *Bambusa oldhamii* (BO), the AGR was 0.03, 0.08 and 0.05 for the control, raw slurry and centrifuged slurry treatment plot, respectively. For the *species Bambusa vulgaris* (BVV), the AGR of shoots was 0.02, 0.05 and 0.03 the control, raw slurry and centrifuged slurry treatment, respectively. The shoot AGR was higher for the raw slurry treatment plot from 46%, 111% and 188% for the species BO, BT and BVV, respectively, compared to the control. For the centrifuged slurry treatment plot, the shoots AGR was higher from 61%, 94%, 139% for the species GW, BVV and BO, respectively, compared to the control plot. The species GW and BVV showed a higher AGR for the raw slurry treatment than with the centrifuged slurry treatment. On the contrary the species BO showed a higher AGR with the centrifuged slurry treatment than with the raw slurry treatment.

All species showed an increase of the mean shoot diameter with the raw and centrifuged slurry treatment, compared to the control (*p <* 0.001) (Figure 2b). For the species GW the average shoot diameter increased from 16 millimeters for the control, to 25 and 21 millimeters for the raw slurry and centrifuged slurry treatment, respectively. For the species BO, the average shoot diameter increased from 29 millimeters for the control, to 36 and 35 for the raw slurry and centrifuged slurry treatment, respectively. For the BVV species the average shoot diameter increased from 36 millimeters for the control, to 44 and 50 millimeters for the raw slurry and centrifuged slurry treatment, respectively. The average shoot diameter was increased by 24%, 21% and 60% for the species BO, BVV and GW, respectively with the raw slurry treatment, compared to the control and by 21%, 36% and 36% for the species BO, BVV and GW, respectively, with the centrifuged slurry treatment, compared to the control. The mean number of shoot increase differed between the species (*p* < 0.001). The species GW and BO showed larger diameter for the raw slurry treatment than with the centrifuged slurry treatment. On the contrary the species BVV show larger diameter for the centrifuged slurry treatment than with the raw slurry treatment. The species BVV show the largest shoot diameter for all the three treatments.

**Figure 3:**
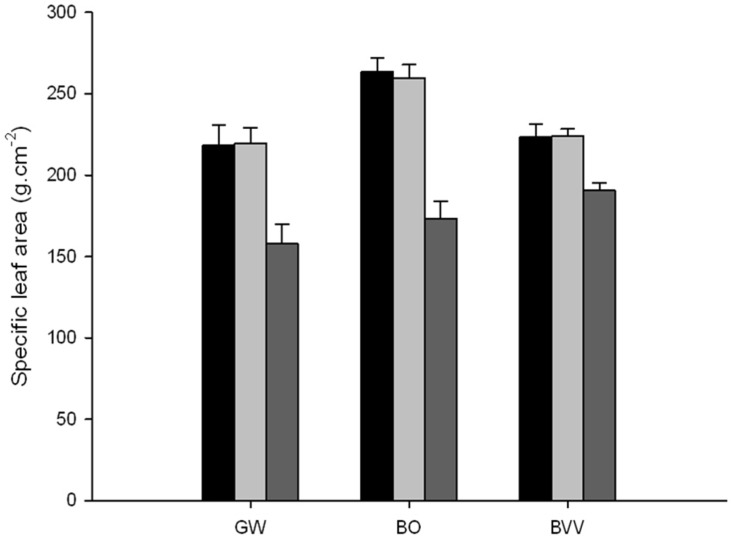
Average specific leaf area (SLA) by species and by treatment plot; *Gigantochloa wrayii* (GW), *Bambusa oldhamii* (BO), *Bambusa vulgaris* (BVV). For each bamboo species: raw slurry, centrifuged slurry and control, from left to right.

The Specific Leaf Area (SLA) increased significantly for the two pig slurry treatments, compared to the control (*p <* 0.001) (Figure 3). For the species GW, the SLA increased from 157 for the control, to 218 and 219 g·cm^−1^ with the raw slurry and centrifuged slurry treatment, respectively. For the species BO the SLA increased from 173 for the control to 223 and 259 g·cm^−1^ with the raw slurry and centrifuged slurry treatment, respectively. For the species BVV the SLA increase from 190 for the control to 223 and 224 g·cm^−1^ with the raw slurry and centrifuged slurry treatment, respectively. On the whole, the increase of SLA is nearly the same for the two pig slurry treatments compare with control and was about 17% for BVV, 38% for GW, 50% for BO.

### 2.2. Nitrogen, Phosphorus and Potassium Contents

The nitrogen content in leaves was significantly increased by the pig slurry treatment (*p <* 0.001) (Table 1). On the whole, the average nitrogen content in leaves increased from 26 g·kg^−1^ Dry Matter (DM) for the control to 33.5 and 33.6 g·kg^−1^ DM with the raw slurry and centrifuged slurry treatment, respectively. The increase in nitrogen was about 18%, 25%, 28% for the species BO, BVV and GW, respectively, with the centrifuged slurry and 11%, 25% and 35% for the species BO, BVV and GW, respectively, with the raw slurry treatment. The species BVV showed the highest nitrogen content in leaves with 34.5 and 35 g·kg^−1^ DM for the raw slurry and centrifuged slurry treatment, respectively. The species GW showed higher nitrogen content with the raw slurry than with the centrifuged slurry treatment, i.e., 29.8 and 28.4 g·kg^−1^ DM, respectively. On the contrary, the BO species showed higher nitrogen content in leaves with the centrifuged slurry than the raw slurry treatment, i.e., 31.9 and 29.9 g·kg^−1^ DM, respectively.

### 2.3. Aboveground Biomass Yield and Nutrient Uptake

The species irrigated with raw slurry showed 3.6, 3.7- and 8.2-times higher nitrogen stored in bamboo biomass than the control, for the species BVV, BO and GW, respectively (Table 2). For the species irrigated with centrifuged slurry, the total nitrogen stored was 3.2, 3.4- and 10.5-times higher compared to the control for the species BVV, BO and GW, respectively.

The species irrigated with raw slurry showed 0.4, 1.8- and 2.8-times higher total phosphorus stored in bamboo biomass than the control for the species BVV, BO and GW, respectively. For the species irrigated with centrifuged slurry the total phosphorus stored was 0.4, 0.9- and 1.3-times higher for the species BVV, BO and GW, respectively.

The species irrigated with raw slurry showed 2.2, 3.0- and 7.6-times higher total potassium stored in bamboo biomass than the control for the species BVV, BO and GW, respectively. For the species irrigated with centrifuged slurry the total potassium stored was 1.9, 3.7- and 4.7-times higher for the species BVV, BO and GW.

The species irrigated with raw slurry showed 1.9, 1.9- and 5.4-times higher carbon stored in bamboo biomass than the control for the species BVV, BO and GW, respectively. For the species irrigated with centrifuged slurry the total carbon stored was 1.9, 2.2- and 3.9-times higher for the species BVV, BO and GW, respectively.

The highest nitrogen content stored in plant parts was found for the species BVV with 1621.6 kg·ha^−1^ for the raw slurry treatment, followed by the species BVV with 1225.4 kg·ha^−1^ for the raw slurry treatment and GW with 864.6 kg·ha^−1^ for the raw slurry treatment (Table 3). The highest phosphorus content stored in plant parts was found for the species BO with 269.6 kg·ha^−1^ for the raw slurry treatment, followed by species BVV with 225.5 kg·ha^−1^ for the control and GW with 85.2 kg·ha^−1^ for the raw slurry treatment. The highest potassium content stored in plant parts was found for the species BO with 1363.9 kg·ha^−1^ for the centrifuged slurry treatment, followed by species BVV with 1194.7 kg·ha^−1^ for the raw slurry treatment and GW with 563.3 kg·ha^−1^ for the raw slurry treatment. The highest carbon content stored in plant parts was found for the species BVV with 67 kg·ha^−1^ for the centrifuged slurry treatment, followed by the species BO with 41.1 kg·ha^−1^ for the centrifuged slurry treatment and GW with 25.7 kg·ha^−1^ for the raw slurry treatment.

After two years of growth the highest fresh biomass yield was for the species BVV with 256.2 t·ha^−1^, followed by the species BO with 202.2 t·ha^−1^ and the species GW with 106.6 t·ha^−1^. The highest dry biomass yield was for the species BVV with 135.2 t·ha^−1^, followed by the species BO with 95.6 t·ha^−1^ and GW species with 60 t·ha^−1^ (Table 3).

### 2.4. Nitrogen and Phosphorus Balance

In total, nitrogen spread (5254 kg·ha^−1^) with the raw slurry, 2.8% was leached through the soil, 14.6% was uptake by bamboo plantation, 35.7% was retained by the soil and 46.9% represent the nitrogen imbalance. On the total nitrogen spread (4088 kg·ha^−1^) with the centrifuged slurry, 1.4% was leached, 12.6% was uptake by bamboo, 54.8% was retained by soil and 31.2% represent the nitrogen imbalance (Table 4).

On the total phosphorus spread with the raw slurry (1397 kg·ha^−1^), 0.04% was leached through the soil, 7.2% was uptake by bamboo plantation, 53.6% was retained by the soil and 39.2% represent the phosphorus imbalance (Table 5). On the total phosphorus spread with the centrifuged slurry (365 kg·ha^−1^), 0.02% was leached through the soil, 12.8% was uptake by bamboo, 51.6% was retained by the soil and 35.6% represent the phosphorus imbalance.

## 3. Discussion

The species irrigated with pig slurry showed higher *Fv/Fm* values than the species irrigated with tap water. For the species irrigated with pig slurry the *Fv/Fm* values reach 0.840 and 0.818 for the raw and the centrifuged slurry, respectively. These values were close to the maximum value for a plant quantum yield, i.e., 0.842 [33]. These results reveal that all the bamboos were in a growth state during the experiment and were not limited by any stress. All the three curves followed the same variations during the experiment; these variations were caused by the change in environment conditions, especially the air temperature that can affect the photosynthetic system [34,35,36]. At the beginning of the fluorescence measurements (January) the bamboo species planted on the three plots were irrigated only with tap water. During this period the *Fv/Fm* values were similar between species. After the pig slurry supply at high rates (July 2009), an increase of the *Fv/Fm* values was observed for the two pig slurry plots. Between the beginning and the end of the experiment the species’ *Fv/Fm* values increased from 0.699 up to 0.810 for the raw slurry treatment and 0.664 up to 0.815 for the centrifuged slurry treatment. These results indicate an improvement of the photosynthetic apparatus due to the increase in nutrient supply. These results comply with the increase in leaves nitrogen content for the species planted in the two pig slurry treatments compared to the control. Several studies have demonstrated that the photosynthetic activity is positively correlated to the nitrogen content in leaves [37,38]. The species planted on the control plot showed an average nitrogen content in leaves of 26 g·kg^−1^ DM. This result corresponds to the nitrogen content that can be found in a natural bamboo forest [39,40,41]. The nitrogen content for the raw slurry and centrifuged slurry were 33.5 and 33.6 g·kg^−1^ DM, respectively, values that are higher than 3% of the dry mass, the maximum nitrogen content recommended by Kleinhenz et al. [26] for optimal biomass yield of bamboo.

With the pig slurry application, the SLA was also increased whatever the pig slurry form by 17%, 38% and 50% for the BVV, GW and BO species, respectively compared to the control. The SLA and the nitrogen were positively correlated (*p <* 0.05) as observed by many authors [32,38]. All these parameters are related to growth rate; indeed, the application of pig slurry increased the nitrogen content in leaves which in turn increased the chlorophyll *a* content. As a result, the photosynthetic efficiency is improved [38] and a higher amount of carbohydrates are synthesized that promote the culm emergence [42]. These results comply with the increase of the AGR and diameter of shoots with the pig slurry treatments, compared to the control.

On average the AGR has increased by 35.5% and 31.8% for the raw slurry and the centrifuged slurry treatments, respectively, compared to the control. The shoot diameter was increased by 115% and 98% for the raw slurry and the centrifuged slurry treatment, respectively, compared to the control treatment. The increase in the number and the shoots diameter depend on the species and treatment (*p <* 0.05). Since the number of shoots and diameter were increased with the pig slurry supply, the biomass was increased too. The biomass was significantly higher for the species irrigated with the pig slurry than those from the control plot (*p <* 0.001). The species BO and BVV showed higher biomass yield with the centrifuged slurry than with the raw slurry treatment with 202.2 t·ha^−1^ and 256.2 t·ha^−1^, respectively. On the contrary the species GW show a higher biomass yield with the raw slurry treatment than with the centrifuged slurry treatment with 106.6 t·ha^−1^. These results suggest a preference for a form of nitrogen supplied by the type of pig slurry spread. Kleinhenz and Midmore [43] have shown that the NH_4_^+^ form of nitrogen was taken up more effectively than NO_3_^−^ by the species *B. oldhamii.* These results are in contradiction with ours. Indeed, the BO species have shown a higher biomass with the centrifuged slurry which is more concentrate with NO_3_^−^ than NH_4_^+^. The NO_3_^−^ concentration in the centrifuged slurry is higher than in the raw slurry (Table 6) because of the nitrification process occurring during the decantation of the pig slurry in the storage tank [44]. The optimal ratio between NH_4_^+^ and NO_3_^−^ for optimal growth depends on the plant species, environmental conditions, developmental stage and on the concentration of nitrogen supplied [45]. The species BO and species BVV should have prefer the ratio of the centrifuged slurry and the species GW, the raw slurry. These results comply with the nitrogen stored in bamboo leaves (Table 1).

The nitrogen imbalance represents 46.9% and 31.2% for the raw slurry and the centrifugal slurry treatment, respectively (Table 4). This loss of nitrogen out of the system can be explained by the volatilization of pig slurry during spreading and to the nitrogen which was stored into the belowground parts of bamboo (rhizomes and roots). Indeed, Rochette et al. [46] and Chantigny et al. [47] report an average of 30% to 40% loss of nitrogen through ammonia volatilization and the belowground biomass of bamboo was estimated to be 30% to 50% of the aboveground biomass [32,48]. A great part of nitrogen was retained by the soil, i.e., 35.7% and 54.8%. This result can be explained by the high anion exchangeable capacity (AEC) of the different soils found on the Réunion island, which can retain effectively the nitrates [12,49].

The phosphorus imbalance represents 39.2% and 31.2% for the raw slurry and the centrifugal slurry treatment, respectively (Table 5). This part of phosphorus is the phosphorus, which was tightly bound to the soil particles, but only the exchangeable fraction of phosphorus was measured during the experiment. Another part of this phosphorus was taken up in the belowground biomass of bamboo as well.

For a plantation of 1600 clumps/ha, the average dry biomass was 28, 30.4, 66.3 t·ha^−1^·yr^−1^ for the species GW, BO and BVV, respectively for the first year and 27.9, 53.2 and 63.2 t·ha^−1^·yr^−1^ for the second year. These values were higher than those found in literature, Shanmughavel et al. [50] report the highest annual biomass yield from the literature with 49 t·ha^−1^·yr^−1^ for *Bambusa* bamboos. However, such density of plantation (1600 clump/ha) could limit the biomass yield at long term because of the competition for the light.

The carbon stored in bamboo biomass reach 18.9 to 67 t·ha^−1^ in two years, so we can expect an average of 9 à 30 t·ha^−1^·yr^−1^ of carbon stored in bamboo biomass. These values are higher from those reported in literature. Yen et al. [51] report 9.89 t·ha^−1^·yr^−1^ for the species *Phyllosatchys makinoi* hayata and Isagi et al. [52] report 8.5 t·ha^−1^.an^−1^ for the species *Phyllostachys pubescens* J. Houz. but these are values are for temperate species without any fertilization.

The species BVV and BO were the species which stored the highest amount of nitrogen and phosphorus in their biomass. In its aboveground parts, the species BO stored 1225.4 kg·ha^−1^, 269.6 kg·ha^−1^ and 1089.7 kg·ha^−1^ of N, P and K, respectively and BVV species stored 1621.6, 100.8, 1194.7 kg·ha^−1^ of N, P and K, respectively based on five-years-old bamboo species planted with a density of 1600 clumps per hectare. The BVV species seems to be a good candidate for the treatment of pig slurry when taking account his high biomass yield and nitrogen storage. On the other hand, the BO species seems adapted for the wastewater treatment when taking account his high phosphorus uptake compare to the other species.

## 4. Materials and Methods

### 4.1. Experimental Conditions

The experiment was conducted over two years from June 2008 to June 2010, on Réunion Island, an overseas French department in the southwest Indian Ocean. The experimental site was located at the agricultural high school (LEPA) of Saint Joseph (21°22’58” S; 55°36’25” E), at an elevation of 18 m. During the two years of experimentation, the site had a temperature range of 17.5 to 45.3 °C, a rainfall range of 509 to 1443 mm·yr^−1^ and an average relative humidity of 69.3%.

Seven species of clumping bamboo were selected for the experiment: *Bambusa vulgaris* Schrad. (BVV), *Bambusa oldhamii* Munro (BO), *Bambusa multiplex cv. golden goddess* (Lour.) Raeusch., *Bambusa multiplex cv. Alphonse Karr* (Lour.) Raeusch., *Bambusa tuldoides* Munro, *Dendrocalamus asper* (Schult.) Backer and *Gigantochloa wrayi* Gamble (GW). These species were chosen for their high biomass yields and because these species are the most studied in literature [32,53,54,55,56,57]. Before being planted at the experiment site, the bamboos were grown in a nursery. For each species, a cutting of mature culm from a mother clump was taken and planted in soil to allow the sprouting of roots and rhizomes over a one-year period. Grown cuttings were transplanted into 3-liter containers for one year and then into 15-liter containers for a further before being planted in 70-liter containers.

On the experiment site, three field plots of 250 m^2^ were designed and a buffer zone of five meter between each plot was created to avoid any contamination and subsurface transfer of pig slurry between plots. A total of 40 bamboo clumps were planted in each plot at a plantation density of 1600 clumps/ha. Bamboo species were planted in June 2008, that is to say five months before starting the experiment, to allow for the bamboo’s proper rooting. At the beginning of the experiment, bamboo species were three years-old.

The same number of bamboo plants was planted on each plot and were distributed in the same order through the plots. The field experimentation had a sandy loam soil with 60% of sand and 25% of silt which characteristics are listed in Table 2. The soil was an eutric arenosol [58,59] developed from volcanic materials; Réunion Island is a volcanic island.

Two of the three plots were supplied with pig slurry and the last one with tap water to serve as control. Tap water do not have any detrimental impact on the growth of bamboos, as shown in our previously published researches [60,61] and in the study conducted by Jiang et al. [62]. Two forms of pig slurry were used for the experiment, i.e., a “raw slurry” and “centrifuged slurry”. The raw slurry was taken directly from the pig factory near the experiment site, pumped into a storage tank without any pretreatment. The centrifuged slurry was the liquid fraction of the raw slurry obtained by a mechanic solid/liquid separator Bargam B/DF 300 (Bargam S.p.A, Italy). Pig slurries and tap water were spread by four pipelines with seven sprinklers on each line (Nelson irrigation Corp., USA). The lines were fixed at 50 cm above the soil. Each plot was irrigated with 4 mm once a week from October 2008 to July 2009 and with 8 mm twice a week from July 2009 to November 2009 (Table 7). The pig slurry volume was chosen according to the soil water holding capacity (Table 8). The volume was increased from July 2009 to November 2009 to ensure leachates collection in lysimeters. After each spreading, a rinse cycle was done in the spreading system to avoid the sprinkler clogging. The same volume of pig slurry or tap water was spread on each plot, measured with a flow meter.

A total of 67 m^3^ (268 mm) of pig slurry was spread on each plot, the characteristics of which are listed in Table 6. The total nitrogen, phosphorus and potassium supplied were 5.3, 1.4, 5.5 t·ha^−1^, respectively for the raw slurry treatment and 4.1, 0.4, 5.1 t·ha^−1^, respectively for the centrifuged slurry treatment.

The leachates were collected by Passive Capillary Fiberglass Wick Lysimeters. Lysimeters were made of stainless-steel square of 45 cm × 45 cm × 5 cm with a 2 cm diameter hole in the corner to allow a 1.45 cm fiberglass wick to pass through. The lysimeters were filled with soil and the wick was frayed and spread on the lysimeter. The wicks were cut at 60 cm long to match the expected pressure at 100 cm of soil depth [63]. The lysimeters were buried at 100 cm depth without disturbing the upper soil layers. Below each lysimeter a 20-l collector tank was buried at 160 cm to collect leachates from the lysimeter. Two pipes linked to the collector tank allowed the water to be collected by a pump. On each plot, four lysimeters were buried: two lysimeters were buried below two different bamboo species randomly chosen and two lysimeters were buried below interrow of bamboo species randomly chosen.

### 4.2. Soil Analysis

A preliminary soil analysis was performed before the pig slurry supply (Table 8). Three soil samples were taken on each plot with an auger (SDEC France, France) at four depths: 0–30 cm, 30–60 cm, 60–90 cm and 90–120 cm. The three soil samples were mixed by depth and analyzed for nitrogen, carbon, phosphorus and potassium determination. Nitrogen and carbon content were determined using the Dumas method by means of an element analyzer (CN 2000, LECO Corporation, USA). The exchangeable phosphorus was determined by the Olsen–Dabin method [64] by means of the ammonium molybdate spectrometric method (ISO 6878:2004) with a continuous flow colorimeter (Proxima, Alliance Instruments Italy). The CEC and exchangeable potassium were determined by hexamine cobalt chloride extraction [65] and determined by atomic absorption spectrophotometry (220FS, Varian Inc., USA). The pHwater and pHKCl were measured according to the NF ISO 10390 standard at a soil/water volume ratio of 1:5. 

Each month, soil samples were taken following the same protocol as for the preliminary soil analysis and analyzed for NH4-N and NO3-N determination. The soil samples were analyzed by KCl (1-M) extraction at a soil/water volume ratio of 1:5 and assayed by continuous flow colorimeter (Proxima, Alliance Instruments Italy). At the end of the experiment, a final soil analysis was done for total nitrogen and phosphorus determination.

### 4.3. Pig Slurry and Leachates Analysis

Samples of raw and centrifuged slurry were taken every month. The nitrogen content of pig slurry was analyzed by the Kjeldahl method [66] with a digestion unit B-435 and a distillation unit B324 (Büchi Labortechnik AG, Switzerland). The mineral nitrogen (NO_3_^−^ and NH_4_^+^) were analyzed by capillary ion analysis (Waters Corp., USA) [67]. The total phosphorus and potassium content were determined by a preliminary dry combustion (500 °C) and by the ammonium molybdate colorimetric method with a colorimeter for phosphorus (Proxima, Alliance Instruments Italy) and by atomic absorption spectrophotometry (220FS, Varian Inc., USA) for potassium content. The carbon content was determined using the Dumas method by means of an element analyzer (CN 2000, LECO Corporation, USA).

The leachates volumes were collected from the lysimeters every two weeks and measured with a bucket. Each collected sample was taken for nitrogen and phosphorus analysis: the mineral nitrogen (NO_3_^−^ and NH4^+^) and phosphate (HPO_4_^2−^) were analyzed by Capillary ion analysis (Waters Corp., USA).

### 4.4. Growth Measurements and Bamboo Biomass Estimations

Three of the seven species were studied during the two years of experimentation. Three clumps per species of *B. Oldhamii*, *B. vulgaris* and *G. wrayi* were measured in each plot. The number and diameter of shoots produced every month was counted and measured with a digital caliper in order to determine the effect of the pig slurry treatment on the bamboo growth. The average absolute growth rate (AGR) of each species was determined every month according to the Equation (1) [68]:

AGR = n_2_ − n_1_/t_2_ − t_1_ (1)

where n_2_ was the final number of shoots, n_1_ the initial number of shoots and t the time interval between the two counts.

Three sampling campaigns were done during the two years of experimentation to determine the biomass yield and the total nutrient stored in bamboo biomass. The first one was done in December 2008, an intermediate sampling was done in December 2009 and the last one was done in June 2010, at the end of the experiment (two years after planting). In each sampling campaign, all the culms produced during the year were counted. The biomass yield was determined using allometric equations [69]. For each bamboo species, the allometric equations were established using the basal diameter. Three culms per clump were randomly sampled among the culms produced during the year. The basal diameter was measured and the total fresh biomass, fresh leaf biomass, fresh branches biomass and fresh culm biomass were weighed with a 0.1-g-precision scale (Kern & Sohn GmbH, Germany). Subsamples of leaves were taken immediately scanned with a scanner (Mustek Scanexpress, Mustek Systems Inc., Taiwan) and weighted. The leaf area was calculated using scan images processed using Adobe Illustrator CS4 software (Adobe Systems Inc., USA). The specific leaf area (SLA), i.e., leaf area per mass unit, was calculated per clump. Subsamples of leaves and culms (including branches) were taken to determine the dry mass (DM) of each part and for chemical analysis once dried.

Regression equations were established between the fresh mass (y) or the dry mass (y) and the basal diameter (x). Raw data were log-transformed to normalize the data distribution and to linearize the regression functions according to the Equation (2)

Log (y) = a + b log (x) (2)

Regression equations were computed with Minitab 15 software (Minitab Inc., USA). This Equation (1) was transformed to obtain the standard form of the allometric Equation (3) [70]:

y = bx ^a^ (3)

A correction factor was then applied to the final biomass result (y) to correct the bias engendered by the logarithm transformation using the following Equation (4) [71]:

CF = exp ^(SEE^2/2)^ (4)

where CF is the correction factor and SEE the standard error of the estimate of the regression.

The total aboveground biomass produced each year was determined for each species thanks to the allometric equation obtained. At the end of the experiment, all the bamboos were cut from the plot and their total biomass was weighted.

### 4.5. Plant Tissues Analysis

To determine the dry mass, subsamples of leaves, branches and culms were oven-dried at 70 °C for 48 h in a drying oven (Memmert, GmbH & Co, Germany) to preserve the nutrients for chemical analysis. Nitrogen content was determined using the Dumas method by means of an element analyzer (CN 2000, LECO Corporation, USA). For phosphorus, carbon and potassium contents, the same method was used as for the pig slurry. Only the species *B. oldhamii* (BO)*, G. wrayi* (GW)*, B. vulgaris* (BVV)*, D. strictus* (DS) were analyzed.

### 4.6. Chlorophyll A Fluorescence Measurements

Fluorescence measurements were done using a pulse amplitude modulation portable fluorometer (Mini-PAM, Walz GmbH, Germany). All measurements were made on mature leaves from culms produced during the year. The maximum quantum yield of photosystem II (PSII)—noted *F_v_/F_m_* in the following—was obtained by dark-adapting leaves for 20 min, as recommended by Rascher et al. [72], before applying a saturation pulse of 8000 µmol·m^−2^·s^−1^ for 800 milliseconds.

Fluorescence measurements were done before and after each spreading session of pig slurry. The measurements were done in the morning to avoid the diurnal photoinhibition of midday [35,73]. The measurements of *F_v_/F_m_* were performed on three randomly selected leaves in each clump studied. The measurements were done on three clumps per species with three repetitions per clump. We focused on the following species: *B. oldhamii* (BO)*, G. wrayi* (GW)*, B. vulgaris* (BVV) for the fluorescence measurements.

### 4.7. Statistical Analyses

A linear mixed model for repeated measure analysis (SPSS Inc., IBM, USA) was used to investigate the difference between shoots AGR, specific leaf area, fluorescence measurements, shoot diameter and biomass yield among species. Factors included in the model were “species”, “treatment plot”, with the “species” factor nested within the “treatment plot” factor as a fixed effect and the “nested plot*species” factor as a random factor. Sphericity was checked with Mauchley’s test; when this assumption was rejected the Greenhouse–Geisser corrections were used for the F-statistics.

A two-tailed Pearson correlation analysis (SPSS Inc., IBM, USA) was conducted to determine the relationships between specific leaf area and nitrogen content in leaves.

## 5. Conclusions

No adverse effects on the growth of bamboos were observed by the application of high quantities of pig slurry. Pig slurry supply on bamboo plantation increased the photosynthetic rate of bamboo, their specific leaf area, the number and the diameter of shoots produced. All the bamboo species studied produced 1.8 to 6 times more aboveground biomass with the application of pig slurry compare with bamboo species irrigated only with tap water. The species have shown different biomass yield depending on the type of pig slurry supplied. These results suggest a preference for the form of nitrogen supplied by the two types of pig slurry (NH_4_^+^ or NO_3_^−^). The biomass results obtained in this study are to our knowledge the highest biomass yield reported. The pig slurry application has shown little leaching of nitrate, about 1.4% to 2.8% of the total nitrogen applied on the plots. The species *Bambusa vulgaris* and *Bambusa oldhamii* were the most productive bamboo species and seems to be interesting species for wastewater water treatment [60,61].

## Figures and Tables

**Table 1:** Average nutrient content in leaves by treatment (g·kg^−1^ dry matter (DM)). *Gigantochloa wrayii* (GW), *Bambusa oldhamii* (BO), *Bambusa vulgaris* (BVV).

Species	Nutrient	Raw Slurry	Centrifuged Slurry	Control
*G. wrayi*	N	29.9 ± 1.4	28.5 ± 1	22.1 ± 0
	P	1.8 ± 0.2	1.9 ± 0.4	3 ± 0.1
	K	14.3 ± 3.1	10.9 ± 0.8	9.3 ± 1.1
*B. oldhamii*	N	30 ± 2.1	31.9 ± 1.7	26.9 ± 0.5
	P	1.6 ± 0	1.6 ± 0.1	3.6 ± 0.3
	K	12 ± 1.4	9.8 ± 0.8	7.4 ± 1.1
*B. vulgaris*	N	34.5 ± 0.6	35.1 ± 0.6	27.5 ± 1.2
	P	1.7 ± 0	1.9 ± 0	2.4 ± 0.2
	K	12.4 ± 0.5	13.5 ± 0.5	13.5 ± 1

**Table 2:** Total nitrogen, phosphorus, potassium and carbon stored in the aboveground biomass.

Species	Plot	Total Nitrogen (kg·ha^−1^)	Total Phosphorus (kg·ha^−1^)	Total Potassium (kg·ha^−1^)	Total Carbon (t·ha^−1^)
*G. wrayi*	Raw slurry	864.6 ± 78.9	85.2 ± 10.0	563.3 ± 9.3	25.7 ± 2.1
	Centrifuged slurry	478.0 ± 23.8	38.2 ± 2.2	353.4 ± 46.1	18.9 ± 0.8
	Control	82.1 ± 24.0	30.5 ± 8.2	74.6 ± 18.0	4.8 ± 1.4
*B. oldhamii*	Raw slurry	1225.4	269.6	1089.7	35.7
	Centrifuged slurry	1032.8 ± 90.4	130.1 ± 8.8	1363.9 ± 83.5	41.1 ± 2.4
	Control	303.1 ± 60.9	152 ± 42	366.7 ± 43.6	18.4 ± 3.7
*B. vulgaris*	Raw slurry	1621.6 ± 612.0	100.8 ± 49.0	1194.7 ± 273.3	65.1 ± 18.6
	Centrifuged slurry	1355.3 ± 373.2	89.3 ± 26.7	997.2 ± 138.2	67.0 ± 16.6
	Control	417.4 ± 121.3	225.5 ± 99.9	532.1 ± 159.9	34.9 ± 8.2

*Gigantochloa wrayii* (GW), *Bambusa oldhamii* (BO), *Bambusa vulgaris* (BVV).

**Table 3:** Annual aboveground biomass yield, total fresh biomass and total dry biomass at the end of the experiment.

Species	Treatment Plot	Year 1 (t·ha^−1^·yr^−1^)	Year 2 (t·ha^−1^·yr^−1^)	Total Fresh Mass (t·ha^−1^)	Year 1 (t·ha^−1^·yr^−1^)	Year 2 (t·ha^−1^·yr^−1^)	Total Dry Mass (t·ha^−1^)
*G. wrayi*	Raw slurry	64.9	40.9	106.6 ± 4.5	35.4	24.1	60 ± 2.6
	Centrifuged slurry	39.1	61.7	101.3 ± 14.5	20.7	31.7	52.7 ± 7.6
	Control	9.3	7.9	17.8 ± 5.2	5.5	4.5	10.3 ± 3
*B. oldhamii*	Raw slurry	66.8	80.5	152 ± 17.4	31.3	46.6	80.4 ± 9.2
	Centrifuged slurry	69.9	118.8	202.2 ± 11.8	29.5	59.8	95.6 ± 5.6
	Control	16.5	46.2	66.2 ± 13.3	9.7	27.1	38.8 ± 7.8
*B. vulgaris*	Raw slurry	101.4	140.2	243 ± 42.4	55.1	72.9	128.7 ± 22.5
	Centrifuged slurry	158.2	96.9	256.2 ± 47.6	81.1	53.6	135.2 ± 25.1
	Control	26.4	101.1	128.3 ± 30.6	15.1	57.9	73.4 ± 17.5

*Gigantochloa wrayii* (GW), *Bambusa oldhamii* (BO), *Bambusa vulgaris* (BVV).

**Table 4:** Nitrogen balance between the bamboo biomass, soil and leachates.

	Raw Slurry Plot (kg·ha^−1^)	% of Total N Applied	Centrifuged Slurry Plot (kg·ha^−1^)	% of Total N Applied	Control (kg·ha^−1^)
Total N soil accumulation	1877	35.7	2242	54.8	−1104
Total N in leachates	146	2.8	56	1.4	0.131
Average total N in bamboo biomass	769	14.6	515	12.6	253
N imbalance	2462	46.9	1275	31.2	-
Total	5254	100	4088	100	-

**Table 5:** Phosphorus balance between the bamboo biomass, soil and leachates.

	Raw Slurry Plot (kg·ha^−1^)	% of Total P Applied	Centrifuged Slurry Plot (kg·ha^−1^)	% of Total P Applied	Control (kg·ha^−1^)
Total P soil accumulation	749	53.6	188	51.6	2582
Total P in leachates	0.53	0.04	0.07	0.02	0.05
Average total P in bamboo biomass	101	7.2	47	12.8	117
P imbalance	547	39.2	130	35.6	-
Total	1397	100	365	100	-

**Table 6:** Pig slurry characteristics (plants take up nitrogen in the form of NO_3_–N (nitrate) or NH_4_–N (ammonium); TKN, total Kjeldahl nitrogen).

Parameters	Raw Slurry	Centrifuged Slurry
Dry matter (g.L^−1^)	23.8	10.8
Density (g.mL^−1^)	1.014	1.008
pH	7.8	8.2
Organic matter (g.L^−1^)	14.5	5.2
Total C (g.L^−1^)	5.7	2.9
TKN (g.L^−1^)	1.9	1.5
NH_4_-N (g.L^−1^)	1.3	1.1
NO_3_-N (mg.L^−1^)	2.3	26.8
Total P (mg.L^−1^)	521.2	136.1
Total K (mg.L^−1^)	2037.3	1911.6

**Table 7:** Volume of pig slurry, irrigation water and precipitation by month (mm).

Month	Pig Slurry	Irrigation and Rinse Water	Precipitation
October-08	12.0	167.0	24.0
November-08	0.0	140.8	64.5
December-08	11.0	169.0	26.0
January-09	4.0	249.6	40.5
February-09	12.0	154.8	230.0
Marh-09	16.0	168.5	145.7
April-09	16.0	136.8	152.7
May-09	0.0	56.9	312.0
June-09	4.0	51.6	115.5
July-09	43.0	7.0	155.0
August-09	22.0	3.0	72.0
September-09	52.0	7.0	54.0
October-09	56.0	7.0	95.5
November-09	20.0	17.2	69.5
**Total**	268.0	1714.4	2158.3

**Table 8:** Soil characteristics at the beginning of the experiment for all depths.

	Depth (cm)			
	0–30	30–60	60–90	90–120
Soil water holding capacity (mm)	0.67	0.61	0.52	0.53
pH water	6.7	6.9	7.1	8.2
pH KCl	5.3	5.5	5.6	6.7
Organic matter g·100g^−1^	2.0	1.3	0.3	0.2
Organic carbon g·100g^−1^	1.2	0.7	0.2	0.1
C/N ratio	10.6	10.7	10.9	9.9
EC (µS·cm^−1^)	68.3	68.2	58.3	54.8
Total N (g·kg^−1^)	1.1	0.7	0.2	0.1
Available P (mg·kg^−1^)	340.5	211.3	50.1	33.1
Total K (mg·kg^−1^)	273.7	267.8	224.8	260.0
CEC (cmol_(c)_. kg^−1^)	8.2	7.5	5.2	5.7

EC: Electrical Conductivity; CEC: Cationic Exchangeable Capacity.

## References

[1] Sáez J.A., Belda R.M., Bernal M.P., Fornes F. (2016). Biochar improves agro-environmental aspects of pig slurry compost as a substrate for crops with energy and remediation uses. Ind. Crop. Prod..

[2] Hjorth M., Christensen K.V., Christensen M.L., Sommer S.G. (2011). Solid-liquid separation of animal slurry in theory and practice. A review. Agron. Sustain. Dev..

[3] Kizito S., Wu S., Kipkemoi Kirui W., Lei M., Lu Q., Bah H., Dong R. (2015). Evaluation of slow pyrolyzed wood and rice husks biochar for adsorption of ammonium nitrogen from piggery manure anaerobic digestate slurry. Sci. Total Environ..

[4] Christel W., Bruun S., Magid J., Jensen L.S. (2014). Phosphorus availability from the solid fraction of pig slurry is altered by composting or thermal treatment. Bioresour. Technol..

[5] Dion P., Le Bozec S., Schramm W., Nienhuis P.H. (1996). The French Atlantic coasts. Marine Benthic Vegetation. Recent Changes and the Effects of Eutrophication.

[6] Schramm W. (1999). Factors influencing seaweed responses to eutrophication: Some results from EU-project EUMAC. J. Appl. Phycol..

[7] Fangueiro D., Ribeiro H., Vasconcelos E., Coutinho J., Cabral F. (2009). Treatment by acidification followed by solid-liquid separation affects slurry and slurry fractions composition and their potential of N mineralization. Bioresour. Technol..

[8] Cardoso F., Shelton D., Sadeghi A., Shirmohammadi A., Pachepsky Y., Dulaney W. (2012). Effectiveness of vegetated filter strips in retention of Escherichia coli and Salmonella from swine manure slurry. J. Environ. Manag..

[9] Jardé E., Gruau G., Mansuy-Huault L. (2007). Detection of manure-derived organic compounds in rivers draining agricultural areas of intensive manure spreading. Appl. Geochem..

[10] Solecki O., Jeanneau L., Jardé E., Gourmelon M., Marin C., Pourcher A.M. (2011). Persistence of microbial and chemical pig manure markers as compared to faecal indicator bacteria survival in freshwater and seawater microcosms. Water Res..

[11] Aubry C., Paillat J.-M., Guerrin F. (2006). A conceptual representation of animal waste management at the farm scale: The case of the Réunion Island. Agric. Syst..

[12] Feder F., Findeling A. (2007). Retention and leaching of nitrate and chloride in an andic soil after pig manure amendment. Eur. J. Soil Sci..

[13] Payet N., Findeling A., Chopart J.-L., Feder F., Nicolini E., Saint Macary H., Vauclin M. (2009). Modelling the fate of nitrogen following pig slurry application on a tropical cropped acid soil on the island of Réunion (France). Agric. Ecosyst. Environ..

[14] Renault D., Paillat J.-M. (1999). Analyse de la production et de l’utilisation des effluents porcins à Grand-Ilet, localité de l’île de la Réunion (Cirque de Salazie). Rapport.

[15] Naim O. (1993). Seasonal responses of a fringing reef community to eutrophication (Réunion Island, Western Indian Ocean). Mar. Ecol. Prog. Ser..

[16] Semple S. (1997). Algal growth on two sections of a fringing coral reef subject to different levels of eutrophication in Réunion Island. Oceanol. Acta.

[17] Chazottes V., Le Campion-Alsumard T., Peyrot-Clausade M., Cuet P. (2002). The effects of eutrophication-related alterations to coral reef communities on agents and rates of bioerosion (Réunion Island, Indian Ocean). Coral Reefs.

[18] Bayo J., Gómez-López M.D., Faz A., Caballero A. (2012). Environmental assessment of pig slurry management after local characterization and normalization. J. Clean. Prod..

[19] Adams N., Carroll D., Madalinski K., Rock S., Wilson T., Pivetz B. (2000). Introduction to Phytoremediation.

[20] McCutcheon S.C., Schnoor J.L. (2003). Phytoremediation:Transformation and Control of Contaminants.

[21] Vymazal J. (2011). Constructed Wetlands for Wastewater Treatment: Five Decades of Experience. Environ. Sci. Technol..

[22] Thawale P.R., Juwarkar A.A., Singh S.K. (2006). Resource conservation through land treatment of municipal wastewater. Curr. Sci..

[23] Singh S.K., Juwarkar A.A., Pandey R.A., Chakrabarti T. (2008). Applicability of high rate transpiration system for treatment of biologically treated distillery effluent. Environ. Monit. Assess..

[24] Arfi V., Bagoudou D., Korboulewsky N., Bois G. (2009). Initial efficiency of a bamboo grove-based treatment system for winery wastewater. Desalination.

[25] Collin B., Doelsch E., Keller C., Panfili F., Meunier J.-D. (2012). Distribution and variability of silicon, copper and zinc in different bamboo species. Plant. Soil.

[26] Kleinhenz V., Milne J., Walsh K.B., Midmore D.J. (2003). A case study on the effects of irrigation and fertilization on soil water and soil nutrient status, and on growth and yield of bamboo (*Phyllostachys pubescens*) shoots. J. Bamboo Ratt..

[27] Dierick D., Hölscher D., Schwendenmann L. (2010). Water use characteristics of a bamboo species (*Bambusa blumeana*) in the Philippines. Agric. Meteorol..

[28] Scurlock J.M.O., Dayton D.C., Hames B. (2000). Bamboo: An overlooked biomass resource?. Biomass Bioenergy.

[29] Hunter I.R., Wu J. (2002). Bamboo Biomass.

[30] Lobovikov M., Ball L., Paudel S., Guardia M., Piazza M., Ren H., Wu J., Russo L. (2007). World Bamboo Resources.

[31] Omoto S., Marui A., Shinogi Y. (2011). Mulching Effects and the Soil Organic Matter Dynamics in Crushed Bamboo Utilization at the Farmland. J. Fac. Agric. Kyushu Univ..

[32] Kleinhenz V., Midmore D. (2001). Aspects of bamboo agronomy. Advances in Agronomy.

[33] Björkman O., Demmig B. (1987). Photon yield of O_2_ evolution and chlorophyll fluorescence characteristics at 77 K among vascular plants of diverse origins. Planta.

[34] Agata W., Hakoyama S., Kawamitsu Y. (1985). Influence of light intensity, temperature and humidity on photosynthesis and transpiration of Sasa nipponica and Arundinaria pygmaea. Bot. Mag. Shokubutsu Gaku Zasshi.

[35] Kumar R., Pal M., Teotia U.V.S. (2002). Diurnal changes in chlorophyll fluorescence in four species of bamboo. J. Bamboo Ratt..

[36] Van Goethem D., De Smedt S., Valcke R., Potters G., Samson R. (2013). Seasonal, Diurnal and Vertical Variation of Chlorophyll Fluorescence on Phyllostachys humilis in Ireland. PLoS ONE.

[37] Li R., Werger M.J.A., During H.J., Zhong Z.C. (1998). Carbon and nutrient dynamics in relation to growth rhythm in the giant bamboo Phyllostachys pubescens. Plant Soil.

[38] Jin A., Jin X., Song Y., Wang H., Zheng B. (2011). Effect of Fertilization on Photosynthetic Features in Phyllostachys pubescens. Bot. Rev..

[39] Shanmughavel P., Francis K. (1996). Biomass and nutrient cycling in bamboo (*Bambusa bambos*) plantations of tropical areas. Biol. Fertil. Soils.

[40] Shanmughavel P., Francis K. (1997). Balance and turnover of nutrients in a bamboo plantation (*Bambusa bambos*) of different ages. Biol. Fertil. Soils.

[41] Shanmughavel P., Sha L., Zheng Z., Cao M. (2001). Nutrient cycling in a tropical seasonal rain forest of Xishuangbanna, Southwest China. Part 1: Tree species: Nutrient distribution and uptake. Bioresour. Technol..

[42] Li R., Werger M.J.A., During H.J., Zhong Z.C. (1998). Biennial variation in production of new shoots in groves of the giant bamboo *Phyllostachys pubescens* in Sichuan, China. Plant. Ecol..

[43] Kleinhenz V., Midmore D. (2002). Improved management practices for culinary bamboo shoots: Local and export markets: A report for the Rural Industries Research and Development Corporation.

[44] Cunault C., Coquinot Y., Burton C.H., Picard S., Pourcher A.M. (2013). Characteristics and composition of fouling caused by pig slurry in a tubular heat exchanger–Recommended cleaning systems. J. Environ. Manag..

[45] Chaillou S., JK V., JF M.-G., Raper CD J., LT H., JP B. (1991). Expression of characteristics of ammonium nutrition as affected by pH of the root. J. Exp. Bot..

[46] Rochette P., Chantigny M.H., Angers D.A., Bertrand N., Côté D. (2001). Ammonia volatilization and soil nitrogen dynamics following fall application of pig slurry on canola crop residues. Can. J. Soil Sci..

[47] Chantigny M.H., Rochette P., Angers D.A., Massé D., Côté D. (2004). Ammonia Volatilization and Selected Soil Characteristics Following Application of Anaerobically Digested Pig Slurry. Soil Sci. Soc. Am. J..

[48] Mailly D., Christanty L., Kimmins J.P. (1997). Without bamboo, the land dies: Nutrient cycling and biogeochemistry of a Javanese bamboo talun-kebun system. Ecol. Manag..

[49] Feder F., Bochu V., Findeling A., Doelsch E. (2015). Repeated pig manure applications modify nitrate and chloride competition and fluxes in a Nitisol. Sci. Total Environ..

[50] Shanmughavel P., Peddappaiah R.S., Muthukumar T. (2001). Biomass production in an age series of *Bambusa bambos* plantations. Biomass Bioenergy.

[51] Yen T.-M., Ji Y.-J., Lee J.-S. (2010). Estimating biomass production and carbon storage for a fast-growing makino bamboo (*Phyllostachys makinoi*) plant based on the diameter distribution model. Ecol. Manag..

[52] Isagi Y., Kawahara T., Kamo K., Ito H. (1997). Net production and carbon cycling in a bamboo *Phyllostachys pubescens* stand. Plant Ecol..

[53] Tripathi S.K., Singh K.P. (1996). Culm recruitment, dry matter dynamics and carbon flux in recently harvested and mature bamboo savannas in the Indian dry tropics. Ecol. Res..

[54] Singh A.N., Singh J.S. (1999). Biomass, net primary production and impact of bamboo plantation on soil redevelopment in a dry tropical region. Ecol. Manag..

[55] Castaneda-Mendoza A., Vargas-Hernandez J., Gomez-Guerrero A., Valdez-Hernandez J.I., Vaquera-Huerta H. (2005). Carbon accumulation in the aboveground biomass of a *Bambusa oldhamii* plantation. Agrociencia.

[56] Kibwage J.K., Netondo G.W., Odondo A.J., Oindo B.O., Momanyi G.M., Jinhe F. (2008). Growth performance of bamboo in tobacco-growing regions in South Nyanza, Kenya. Afr. J. Agric. Res..

[57] Liu K.C., Lin T.S., Lin C.H., Lo H.F., Tsao S.J. (2008). Growth and shoot emergence of green bamboo (*Bambusa oldhamii* Munro) under different temperatures. Acta Hortic..

[58] Feder F. (2013). Soil map update: Procedure and problems encountered for the island of Réunion. Catena.

[59] IUSS Working Group WRB (2014). World Reference Base for Soil Resources 2014. International soil classification system for naming soils and creating legends for soil maps. World Soil Resources Reports No. 106.

[60] Piouceau J., Bois G., Panfili F., Anastase M., Dufossé L., Arfi V. (2014). Effects of High Nutrient Supply on the Growth of Seven Bamboo Species. Int. J. Phytoremediation.

[61] Piouceau J., Panfili F., Bois G., Anastase M., Dufossé L., Arfi V. (2014). Actual evapotranspiration and crop coefficients for five species of three-year-old bamboo plants under a tropical climate. Agric. Water Manag..

[62] Jiang L., Shi G., Ding Y., Lou L., Cai Q. (2013). Differential responses of two bamboo species (*Phyllostachys auresulcata* ‘Spectabilis’ and *Pleioblastus chino* ‘Hisauchii’) to excess copper. Bioenergy Res..

[63] Knutson J.H., Selker J.S. (1994). Unsaturated Hydraulic Conductivities of Fiberglass Wicks and Designing Capillary Wick Pore-Water Samplers. Soil Sci. Soc. Am. J..

[64] Dabin B. (1967). Application des dosages automatiques à l’analyse des sols. 3e partie. Méthode Olsen modifiée In Cahiers ORSTOM Pédologie. Série Pédologie.

[65] Ciesielski H., Sterckeman T., Santerne M., Willery J.P. (1997). Determination of cation exchange capacity and exchangeable cations in soils by means of cobalt hexamine trichloride. Eff. Exp. Cond. Agron..

[66] Kjeldahl J. (1883). A new method for the determination of nitrogen in organic bodies. Anal. Chem..

[67] Saad B., Wei Pok F., Sujari A.N.A., Idiris Saleh M. (1998). Analysis of anions and cations in drinking water samples by Capillary Ion Analysis. Food Chem..

[68] Brown A.H., Johnson Z.B., Chewning J.J., Brown C.J. (1988). Relationships among absolute growth rate, relative growth rate and feed conversion during postweaning feedlot performance tests. J. Anim. Sci..

[69] Shanmughavel P., Francis K. (2001). Physiology of Bamboo.

[70] Navar J., Momba M., Bux F. (2010). Measurement and and assessment methods of forest aboveground biomass: A literature review and the challenges ahead. Biomass.

[71] Sprugel D.G. (1983). Correcting for bias in log-transformed allometric equations. Ecology.

[72] Rascher U., Liebig M., Lüttge U. (2000). Evaluation of instant light-response curves of chlorophyll fluorescence parameters obtained with a portable chlorophyll fluorometer on site in the field. Plant. Cell Environ..

[73] Fernandez-Baco L., Figueroa M.F., Luque T., Davy A.J. (1998). Diurnal and seasonal variations in chlorophyll a fluorescence in two Mediterranean-grassland species under field conditions. Photosynthetica.

